# ENDOSCOPIC SURGERY FOR TREATING SPINAL STENOSIS: AN INTEGRATIVE REVIEW OF RANDOMIZED CLINICAL TRIALS

**DOI:** 10.1590/1413-785220243206e278913

**Published:** 2025-01-10

**Authors:** Rafael Augusto da Silva Aparício, Rodrigo Freitas Maringoni de Oliveira, Felipe de Almeida Pinto, Carlos Gorios, Lorran Azevedo Zanon, Alessandra Masi

**Affiliations:** 1. Hospital Geral de Carapicuiba, Serviço de Ortopedia e Traumatologia, Carapicuiba, São Paulo, SP, Brazil.

**Keywords:** Spine, Stenosis, Endoscopic Surgery, Minimally Invasive Surgery, Coluna Vertebral, Estenose, Cirurgia Endoscópica, Cirurgia Minimamente Invasiva

## Abstract

Background: Spinal stenosis refers to the narrowing of the spinal canal which can generate clinical symptoms secondary to the spinal cord injury itself, or even root involvement. The traditional open surgical procedure to correct spinal stenosis is highly traumatic and risky, and with the development of surgical techniques, endoscopic procedures have been widely used in treating said stenosis, achieving good results with minimally invasive management. Aim: To conduct a literature review regarding endoscopic techniques for correcting spinal stenosis. Method: This is an integrative literature review that surveyed the PUBMED database using the following search strategy: spinal[title] AND stenosis[title] AND surgery[title]. Only randomized clinical trials published in the last 10 years were included in the sample. Results: A total of 13 articles were identified that met the previously established search strategy, all of which were included in the review. Conclusion: The reviewed studies showed that endoscopic surgery to correct spinal stenosis could offer adequate decompression of neural elements, resulting in shorter hospital stays, faster recovery and favorable operative results. *Level of Evidence IV, evidence from descriptive (non-experimental) or qualitative studies.*

## RESUMO

Introdução: O termo estenose na coluna vertebral se refere ao estreitamento do canal vertebral, que por sua vez pode gerar sintomas clínicos secundários à própria lesão medular, ou mesmo ao comprometimento radicular. O procedimento cirúrgico aberto tradicional para correção da estenose vertebral é altamente traumático e arriscado, e com o desenvolvimento das técnicas cirúrgicas, os procedimentos endoscópicos têm sido amplamente utilizados no tratamento da referida estenose, alcançando bons resultados com manejos minimamente invasivos. Objetivo: Realizar uma revisão da literatura a respeito das técnicas endoscópicas para correção da estenose vertebral. Método: Trata-se de uma revisão integrativa da literatura que utilizou como fonte a base de PUBMED, utilizando a seguinte estratégia de busca: spinal[title] AND stenosis[title] AND surgery[title]. Apenas ensaios clínicos randomizados publicados nos últimos 10 anos foram incluídos na amostra. Resultados: Foram identificados 13 artigos que atenderam à estratégia de busca previamente estabelecida, todos incluídos na revisão. Conclusão: Os trabalhos revisados apontaram que a cirurgia endoscópica para correção da estenose espinal poderia oferecer uma descompressão adequada dos elementos neurais, resultando em menor tempo de internação hospitalar, recuperação mais rápida e resultados operatórios favoráveis. *Nível de Evidência IV, evidências de estudos descritivos (não-experimentais) ou com abordagem qualitativa.*

## INTRODUCTION

 Spinal stenosis refers to the narrowing of the spinal canal which in turn can generate clinical symptoms secondary to the spinal cord injury or even root involvement. It can involve the cervical, thoracic (rarely), or lumbar spine, presenting as monosegmental or multisegmental (adjacent or not) and unilateral or bilateral. Cervical or thoracic spinal stenosis can cause root and spinal cord compression, resulting in pain, radiculopathy, myelopathy, or myeloradiculopathy. Lumbar spinal stenosis (LSE), the most common of the three, causes only root compression with typical complaints of neurogenic claudication or radicular leg pain. ^1^

 LSE incidence is four times higher than that of cervical stenosis, totaling five cases per 100,000 individuals, and both coexist in 5% of patients. Up to 14% of patients who seek specialized care for low back pain have spinal stenosis and some degree of the disease is present in up to 80% of patients over 70 years old, when evaluated by imaging techniques. Distinction between narrowing of the canal and stenotic symptoms should be emphasized, as one-fifth of asymptomatic individuals over 60 years old show stenosis on MRI. ^1^ ^,^ ^2^

 Spinal stenosis diagnosis is based on the patient’s history and confirmed by imaging tests. However, physical examination is often not useful in recognizing LES, and neurophysiological studies can help confirm a coexisting pathology or aid in the differential diagnosis, particularly for polyneuropathies of various causes. Additionally, clinical presentation may be complex in a minority of patients with cervical and lumbar stenosis. ^1^ ^,^ ^3^

 Knowledge of the natural history of the disease is fundamental in determining the most appropriate course of treatment. As significant spontaneous improvement is unlikely to occur, intolerable symptoms warrant a more aggressive treatment strategy. Progressive disease is expected for both cervical and lumbar stenosis, probably more dramatically in the former, so the treatment goal should be primarily to halt progression. Nonoperative management includes: exercises, girdles, analgesics, and physical therapy. However, bed rest is no longer considered a treatment alternative. Spinal stenosis surgery is indicated for patients who have been severely symptomatic for a reasonable period of time (most authors consider at least three months). Its biomechanical goal is to relieve pressure on both the spinal cord/thecal sac and nerve roots without causing instability. The clinical goal is to provide pain relief and prevent disease progression by allowing for reversal of neurological deficits if present. ^1^ ^,^ ^4^

 In certain cases, and due to the specific anatomical features of the spine, surgery is the only effective treatment; however, the traditional open procedure is highly traumatic and risky, despite being considered the gold standard for treating spinal stenosis. With the development of surgical techniques, endoscopic technology (represented by the transforaminal endoscopic surgical system) has been widely used to treat vertebral diseases, achieving good results with minimally invasive management since the beginning of the last decade. Based on this, national and foreign specialists have been using endoscopic techniques to treat spinal stenosis routinely. ^4^ ^,^ ^5^

Given this context, this integrative review synthesizes the results of randomized clinical trials that described spinal stenosis treatment with endoscopic surgical techniques.

## MATERIAL AND METHOD

 This is an exploratory study integrative literature review with evidence synthesis. Based on Souza et al., ^6^ the review was divided into the following six steps: 1 - elaboration of guiding question; 2 - bibliographic search or sampling; 3 - data collection; 4 - critical analysis of included studies; 5 – discussion of results; and 6 – presentation of the integrative review. Fulfilling the first step, we formulated the following guiding question: “What are the main endoscopic methods for treating spinal stenosis?” 

 For the bibliographic search for sampling, we surveyed the PUBMED database using the following search strategy: *spinal[title] AND stenosis[title] AND surgery[title]* . Only clinical trials published in the last 10 years were included in the review. 

## RESULTS

Data collection took place in September 2023. Initial search identified 13 studies that met the previously established strategy. Continuing with the critical analysis of the included studies, the titles and abstracts were read resulting in no article excluded.

## DISCUSSION

In this section we discuss the review results by presenting each of the selected articles individually, compiling their proposals, methodology, main results and conclusions. The articles are presented in chronological order based on publication year.

 Usman et al. ^7^ evaluated the feasibility and efficacy of a new minimally invasive spinal surgery technique for LES correction involving a unilateral approach to bilateral decompression. For this purpose, they conducted a single-center cross-sectional observational study from January to December 2010. The study included 60 patients with LES who were randomized to undergo either conventional laminectomy (30 patients, Group A) or unilateral endoscopic approach (30 patients, Group B). Clinical outcomes were measured using the Finneson and Cooper scale. All data were collected using *pro forma* . Different parameters were evaluated for a minimum follow-up period of three months, and were analyzed by descriptive statistics using SPSS version 17. Adequate decompression was achieved in all patients. Compared with the subjects in the conventional laminectomy group, those who received the new procedure (unilateral approach) had a reduced average hospital stay, a faster recovery rate and most patients (88.33%) had an excellent to reasonable operative outcome, according to the Finneson and Cooper scale. Five major complications occurred in all patient groups: two had unintentional dural rupture, two had wound dehiscence, and the fifth patient had worsening symptoms. There was no mortality in the series. According to the authors, the ultimate goal of the unilateral endoscopic approach to treat LES was to achieve adequate decompression of the neural elements. An additional benefit of a minimally invasive approach was the adequate preservation of vertebral stability, as it required only minimal muscle trauma, preserved the supraspinatus/infraspinatus ligament complex and the spinous process; therefore, it allowed for early mobilization. This also shortened the length of hospital stay, reduced postoperative back pain, and led to satisfactory results. 

 Delitto and collaborators ^8^ compared surgical decompression with physical therapy (PT) for LES, also evaluating the differences between genders. They conducted a multicenter randomized controlled trial with LES surgery candidates aged 50 years or older who were randomized to endoscopic surgical decompression or PT. Primary outcome was the physical function score on the *Short Form-36 Health Survey* after two years, evaluated by masked testers. The study took place from November 2000 to September 2007 and had a total of 169 participants who were randomly assigned and stratified by surgeon and gender (87 for surgery and 82 for PT), with a 24-month follow-up completed by 74 and 73 participants in the surgery and PT groups, respectively. Mean improvement in physical function for the surgery and PT groups was 22.4 and 19.2, respectively, and intention-to-treat analyses revealed no difference between the groups. Sensitivity analyses using causal effects methods to explain the high proportion of crossovers from PT to surgery (57%) showed no significant differences in physical function between the groups. As a limitation, the researchers stated that without a control group it was not possible to assess success attributable to any of the interventions. According to the authors, endoscopic surgical decompression produced effects similar to a PT regimen among patients with LES who were candidates for surgery. Additionally, patients and healthcare providers should participate in shared conversations for decision-making that includes full disclosure of evidence involving surgical and nonsurgical LES treatments. 

 Marchand et al. ^9^ commented that in LES, although the benefits of surgery outweighed those of conservative approaches, physical rehabilitation could be used to improve function and minimize the risk of persistent dysfunction. Within this context, the group developed a study protocol to establish the feasibility of a large-scale randomized clinical trial and to evaluate the efficacy of an active preoperative intervention program in improving clinical parameters and functional physical capacity in patients undergoing endoscopic surgery for LES. To this end, 40 patients were recruited and randomly allocated to one of two treatment arms: a six-week supervised preoperative rehabilitation program (experimental group) or standard hospital preoperative management (control group). The intervention group trained three times a week, with each session aimed at improving strength, muscular endurance, spine stabilization, and cardiovascular fitness. Exercise intensity and complexity were gradually increased throughout the sessions, depending on the individual progress of each participant. Primary outcomes were the level of lumbar disability and the level of pain. Secondary outcomes included the use of analgesics, quality of life, patient’s global impression of change, endurance of the lumbar extensor muscles, maximal voluntary contraction of the lumbar flexor and extensor muscles, maximal voluntary contraction of the knee extensors, active lumbar ranges of motion, walking skills and cardiovascular capacity. Primary and secondary outcomes were measured at baseline, at the end of the training program (six weeks after baseline assessment for control participants), and at six weeks, three, and six months postoperatively. According to the authors, their study informed the design of a future large-scale trial on the topic. Additionally, improvements in patients’ physical performance before undergoing endoscopic lumbar surgery could limit the functional restrictions that occurred after a surgical intervention. The results of this study would provide the opportunity to efficiently improve spine care and advance the knowledge of favorable preoperative strategies to optimize postoperative recovery. 

 Försth et al. ^10^ randomly assigned 247 patients between 50 and 80 years of age who had LES at one or two adjacent vertebral levels to undergo endoscopic decompression surgery plus fusion surgery (fusion group) or endoscopic decompression surgery (decompression group). Randomization was stratified according to the presence of preoperative degenerative spondylolisthesis (in 135 patients) or its absence. Outcomes were assessed using patient-reported outcome measures, a six-minute walk test, and an economic health assessment. Primary outcome was the score on the *Oswestry Disability Index* (ODI; ranging from 0 to 100, with higher scores indicating more severe disability) two years after surgery. Primary analysis, which was a per-protocol analysis, excluded the 14 patients who did not receive the assigned treatment and the five who were lost to follow-up. No significant difference was observed between the groups regarding the mean ODI score after two years (mean of 27 in the fusion group and 24 in the decompression group) or the six-minute walk test results (mean of 397 m in the fusion group and 405 m in the decompression group). Results were similar between patients with and without spondylolisthesis and between patients who had five years of follow-up and were eligible for inclusion in the five-year analysis. There were no significant differences between the groups in clinical outcomes. Mean length of hospital stay was 7.4 days in the fusion group and 4.1 days in the decompression group. Operation time was longer, the amount of bleeding was higher, and surgical costs were higher in the fusion group than in the decompression group. During a mean follow-up of 6.5 years, additional lumbar spine surgery was performed in 22% of patients in the fusion group and in 21% of those in the decompression group. Among patients with LES, with or without degenerative spondylolisthesis, endoscopic decompression surgery plus fusion surgery did not result in better clinical outcomes at two and five years than endoscopic decompression surgery alone. 

 Kesänen et al. ^11^ evaluated the impact of preoperative knowledge on anxiety, health-related quality of life (HRQoL), disability, and pain in patients with spinal stenosis treated via endoscopic surgery. For this purpose, 100 patients were randomized into an intervention group (IG, n = 50) or a control group (CG, n = 50). Both groups received routine preoperative education and the IG also underwent a feedback session based on a knowledge test. Primary outcome was anxiety at the time of surgery. HRQoL, disability, and pain were the secondary outcomes measured during a six-month follow-up. IG showed a significant reduction in anxiety after the intervention, whereas in the CG anxiety was reduced only after surgery. Both groups had significant improvement in HRQoL, disability, and pain at the six-month follow-up, but without statistically significant differences between the groups. According to the authors, a higher level of knowledge could reduce preoperative anxiety, but it did not seem to affect self-reported clinical surgery outcomes. 

 Kang and collaborators ^12^ evaluated the feasibility of spinal decompression using the biportal/endoscopic technique compared with microscopic surgery. To this end, 70 patients with LES who underwent laminectomy were included in this study. A numerical table was used to randomize the subjects into two groups: a biportal/endoscopic technique group (BG, n = 36) and a microscopic surgery group (OG, n = 34). A surgeon performed the biportal/endoscopic technique or microscopic decompression with a tubular retractor, depending on the group to which the patient was randomized. Perioperative data and clinical outcomes in the six-month postoperative period were collected and analyzed. Demographics and level of surgery were comparable between the two groups, with shorter operation time (mean 36 ± 11 *versus* 54 ± 9 min), lower Hemovac drain flow rate (mean 25.5 ± 15.8 *versus* 53.2 ± 32.1 ml), lower opioid use (mean 2.3 ± 0.6 *versus* 6.5 ± 2.5 T) and shorter hospital stay (mean 1.2 ± 0.3 *versus* 3.5 ± 0.8 days) shown in the BG group. However, this group did not present significant differences in clinical results compared to OG and favorable clinical results were observed six months after surgery in both groups. Lumbar decompression surgery using the biportal/endoscopic technique presented favorable clinical results, less pain, and shorter hospital stay compared with microscopic surgery in patients with LES. 

 Kesänen et al. ^13^ evaluated the effect of a specific approach to preoperative education (Knowledge Test Feedback Intervention - KTFI) in the verbal and visual comprehension of patients about endoscopic spinal surgeries. They conducted a randomized clinical trial with an intervention group (n = 50) that underwent KTFI and routine education, whereas the control group (n = 50) received only routine patient education. Written description of the surgical procedure and incision design were used as outcome measures at baseline, hospitalization, and discharge at three and six months after surgery. At baseline, half of the participants demonstrated verbal and visual understanding of their surgery and during follow-up, comprehension improved significantly, with no statistically significant differences between the groups. According to the authors, the understanding of patients with spinal stenosis about the surgical procedure was imperfect and educators would need to ensure patient learning by evaluating their comprehension results. 

 Marchand and collaborators ^14^ evaluated the feasibility of conducting a preoperative intervention program with LES patients and reported on the management of the proposed intervention. Patients were allocated to a six-week supervised preoperative rehabilitation program or to a control group. The intervention included supervised exercise sessions aimed at improving the subjects’ strength, muscular endurance, and spinal stabilization. Outcomes were measured at baseline, six weeks before and again six weeks after, three months, and six months after endoscopic surgery. 65% of the eligible participants agreed to participate in the study, of which 5% withdrew before the end of the intervention period. 88% of the possible training sessions were conducted without adverse events. Improvements were observed in favor of the experimental group in the preoperative assessment for active ranges of motion, leg pain intensity, lumbar extensor muscle endurance, and walking capabilities. According to the authors, the results showed that small modifications in the choice of outcome measures would increase the viability of the main study, which has already been discussed in our study. Absence of adverse events, along with positive changes observed in the outcome dependent measures, would warrant conducting a large-scale trial evaluating the efficacy of this type of intervention. 

 Lee et al. ^15^ evaluated, through a prospective randomized study, the impact of posterior epidural adipose tissue (PAT) on the postoperative outcome of lumbar endoscopic decompression surgery for LES, verifying whether PAT was removed or preserved during the surgical procedure. Of the 185 eligible patients selected for the study, 181 patients were enrolled and randomly allocated to group A (PAT removal, n = 90) or group B (PAT retention, n = 91). Primary outcome was pain intensity in the lower back and lower extremities. Secondary outcomes were functional outcomes based on ODI and distance covered, complications during the surgical procedure, and surgical outcomes. Intensity of postoperative pain in the lumbar region and lower limbs was higher in group A than in group B. Functional status in the ODI and in the distance covered was also worse in group B than in group A (64.9% in group A and 66.2% in group B). The number of patients with worsening pain intensity and deterioration of functional status in the postoperative follow-up was significantly higher in group A than in group B. There were no significant differences in surgical outcome and complications between the groups. According to the authors, retention of epidural fat could be favorable in the postoperative results of posterior endoscopic decompression surgery for LES compared with its removal. 

 Marchand and collaborators ^16^ re-evaluated the effectiveness of a preoperative exercise-based intervention program compared with usual care in improving the clinical status, physical capacities, and postoperative recovery of patients waiting for LES endoscopic surgeries. To this end, 68 participants were randomized to receive a six-week supervised exercise-based pre-habilitation program or usual hospital care. Results included clinical and physical measures and data collection occurred at the post-intervention and at six weeks, three and six months after surgery. Significant but small improvements were found in favor of the experimental group in the post-intervention assessment for pain intensity, LES-related disability, lumbar strength in flexion, lumbar extensor muscle endurance, total ambulation time, and sit-to-stand performance. A significant difference in favor of the intervention group was found from the three-month postoperative follow-up for lumbar region-related disability and no adverse events were reported. According to the authors, exercise-based pre-habilitation did not improve the short-term postoperative recovery of patients with LES. 

 Yüce et al. ^17^ compared epidural hemostasis in minimally invasive surgeries for spinal stenosis by fat grafting *versus* gelatin sponge. They conducted an *in vivo* prospective randomized controlled trial in humans, with 24 operated levels of LES being evaluated in two groups: Group A (control group: gelatin sponge) and Group B (experimental group: fat graft). International Normalized Ratio and Prothrombin Time were evaluated in the preoperative period, and the number of hemostatic cotton forceps and systolic and diastolic blood pressure were evaluated intraoperatively. The epidural hemorrhage area, spinal cord size, and the ratio of epidural hemorrhage area to spinal cord size were assessed by an early postoperative lumbar MRI. Mean area of epidural hemorrhage in groups A and B was 1.3±0.5 and 1.2±0.6 cm ^2^ , respectively, and the mean size of the spinal cord was 1.2±0.6 and 1.8±0.6 cm ^2^ on postoperative lumbar axial magnetic resonance imaging. The two groups did not differ significantly regarding the ratio of epidural hemorrhage/spinal cord size or number of intraoperative hemostatic forceps. According to the authors, autologous fat grafting ensured sufficient and safe epidural hemostasis without serious adverse events in minimally invasive spinal surgery, and was preferable because the autologous tissue was harvested easily and quickly. Additionally, the participating surgeons felt safe with this technique and did not require external hemostatic agents. 

 Yagi and collaborators ^18^ developed and validated a machine learning model to predict the postoperative outcome of endoscopic decompression surgeries for patients with LES. They conducted a retrospective multicenter study with 848 patients who underwent decompression surgery for LES at three different medical centers, assessing their HRQoL at the beginning of the study and two years after surgery. Subjects were randomly assigned in a 7:3 ratio to a model-building cohort and a test cohort to assess the accuracy of the models. Twelve predictive algorithms using 68 preoperative factors were used to predict each domain of the Japanese Orthopaedic Association Back Pain Assessment Questionnaire (QADC) and visual analogue scale (VAS) scores at two years postoperatively. The final predictive values were generated using a set of the top five algorithms in prediction accuracy. Correlation coefficients of the main algorithms for each domain established using the preoperative factors were excellent (correlation coefficient: 0.95-0.97 [relative error: 0.06-0.14]) and the performance assessment of each QADC domain and VAS score by the set of five main algorithms in the test cohort was favorable (mean absolute error [MAE] 8.9-17.4, median difference [MD] 8.1-15.6/100 points), with the highest accuracy for mental status (MAE 8.9, MD 8.1) and the lowest for numbness in the glutes and legs (MAE 1.7, MD 1.6/10 points). A strong linear correlation was observed between predicted and measured values (linear correlation 0.82-0.89), while 4% to 6% of individuals had predicted values greater than ±3 standard deviations from the MAE. According to the authors, a machine learning model was successfully developed to predict the postoperative outcomes of endoscopic decompression surgeries for patients with LES using subject data from three different institutions. They also stated that thorough analyses for subjects with deviations from the actual measured values could further improve the predictive probability of this model. 

 Finally, Minetama et al. ^19^ evaluated whether branched-chain amino acids (BCAAs) plus vitamin D supplementation could attenuate loss of muscle mass and strength, accelerate the return of functional mobility, and improve clinical outcomes after endoscopic lumbar surgery for LES. A randomized, blinded, single-center clinical trial was conducted with eighty patients who underwent endoscopic lumbar surgery for LES. Primary outcome was the Zurich claudication questionnaire (ZCQ), and secondary outcomes included knee muscle strength, muscle mass measured by bioelectrical impedance analysis, gait speed, and timed up-and-go test (TUG) at 12 weeks postoperatively. Follow-up assessment was performed for the ZCQ at 52 weeks postoperatively. Patients ingested supplementation (BCAA group: BCAA plus vitamin D and amino acid-free group) twice daily for three weeks from the day after surgery, and received two hours of postoperative inpatient rehabilitation five times a week. No significant differences were observed regarding mean changes in ZCQ between the two groups at 12 weeks and 52 weeks postoperatively. Two weeks after surgery, the group without amino acids showed significant deterioration in knee extensor and flexor strength compared with the BCAA group. At 12 weeks, the BCAA group showed significant improvements in knee extensor strength and knee flexor strength compared with the group without amino acids. There were no significant differences in mean changes in muscle mass, maximum gait speed, and TUG after 12 weeks postoperatively between the two groups. According to the authors, BCAA supplementation with vitamin D did not improve clinical outcomes related to LES after endoscopic lumbar surgery, but muscle strength increased. They stated that future studies should focus on long-term outcomes for muscle mass and physical function, including the development of sarcopenia and frailty. 

## CONCLUSION

Our integrative review revealed that endoscopic surgery to correct spinal stenosis can offer adequate decompression of neural elements, resulting in shorter hospital stays, faster recovery, and favorable operative results. Additionally, the reviewed studies suggest that exercise-based pre-habilitation may be beneficial for some patients but does not have a significant impact on short-term recovery. Decision between endoscopic surgery and other treatment options, such as physical therapy, should be carefully discussed between patients and healthcare professionals considering the available evidence and individual needs. Finally, the use of nutritional supplements such as BCAAs and vitamin D does not seem to have a substantial impact on clinical outcomes after endoscopic lumbar surgery, although patients’ muscle strength increased with the procedure.
